# Body Composition Characteristics of Elite Senior and Under 23 Australian Sprint Kayakers

**DOI:** 10.3390/sports13050143

**Published:** 2025-05-08

**Authors:** Ashleigh G. Keefe, Gyan A. Wijekulasuriya, Amy-Lee M. Bowler, Nicola Bullock, Vernon G. Coffey, Gregory R. Cox

**Affiliations:** 1 Bond Institute of Health and Sport, Faculty of Health Sciences and Medicine, Bond University, Gold Coast, QLD 4226, Australianicola.bullock@ausport.gov.au (N.B.); vcoffey@bond.edu.au (V.G.C.); 2 Institute for Health and Sport, Victoria University, Melbourne, VIC 3011, Australia; gyan.wijekulasuriya@live.vu.edu.au; 3 Maribyrnong Sports Academy Research Centre, Melbourne, VIC 3032, Australia; 4 School of Health, University of the Sunshine Coast, Sunshine Coast, QLD 4556, Australia; abowler@usc.edu.au; 5 Paddle Australia, Gold Coast, QLD 4218, Australia; 6 Australian Institute of Sport, Canberra, ACT 2602, Australia

**Keywords:** body composition, lean mass, dual-energy X-ray absorptiometry, women athletes, paddling, kayak

## Abstract

The aim of this study was to compare body composition characteristics of elite senior and U23 sprint kayak athletes and report body composition changes during the COVID-19-interrupted preparation for the Tokyo 2020 Summer Olympics. A total of 32 Australian kayakers (Men: 20 (Senior = 13, U23 = 7); Women: 12, (Senior = 5, U23 = 7)) undertook body composition assessment using dual-energy X-ray absorptiometry (DXA) from 2017 to 2021. The first DXA assessment for each athlete was used for a cross-sectional analysis to compare senior and U23 sprint kayak athletes. Of the thirty-two kayakers, five senior men kayakers had repeat DXA scans over the data collection period which were used to monitor longitudinal changes in body composition. Senior men kayak athletes were heavier than U23 athletes (*p* = 0.017; 10.4 ± 1.9 kg; *d* = 1.23) but had similar body composition. In contrast, body mass was not different between senior and U23 women kayak athletes (*p* = 0.187), however senior women athletes had a significantly higher lean body mass (LBM; *p* = 0.048; 5.1 ± 1.3 kg, *d* = 1.32) and lower body fat percentage (*p* = 0.011; −4.3 ± 0.8%, *d* = 1.82). The five senior men kayakers exhibited a non-significant decrease in fat mass (*p* = 0.774; 2.9 ± 3.0 kg, *d* = 0.97) and increase in LBM (*p* = 0.234; 2.2 ± 5.9 kg, *d* = 0.38) across the Olympic quadrennial with little change in body mass. Senior men kayak athletes while heavier, have similar body composition compared to their U23 counterparts, whereas senior women kayakers are similar in body mass but differ in body composition compared to their younger counterparts. The relative influence of maturation, specificity of training, or dietary strategies on the observed differences in body composition between senior and U23 men and women kayak athletes are currently unknown and warrant further investigation.

## 1. Introduction

Sprint kayaking is a high-intensity, short duration sport characterized by athletes in a seated position with a double-bladed paddle. Events are differentiated by the number of athletes in the kayak, ranging from one to four, and distance of the event (200 m; 500 m; 1000 m (men only)). Elite sprint kayakers compete for up to ~4 min and possess a combination of high aerobic and anaerobic capacity [1]. For 200 m and 500 m sprint kayak races, the estimated energy contribution is ~50% from the anaerobic systems while 1000 m races are predicted to have ~80% of the energy contribution from the aerobic system [2]. The upper body generates ~80% of stroke force and kayak speed [3], with physical preparation typically focusing on the development of upper body strength, power, and muscular endurance. Upper body musculature is a particularly important physical characteristic for optimal kayaking performance [1,3,4].

Body composition assessment is commonly performed among athlete populations as a means of tracking the amount and distribution of fat free mass (FFM), lean body mass (LBM), and fat mass (FM) [5]. Body composition is associated with key performance measures for kayaking, such as lower body power and upper body strength, and its assessment can be used to evaluate the effectiveness of training periodization and dietary changes [6]. Body composition assessments commonly constitute a part of standard athlete servicing and often include surface anthropometry, bioelectrical impedance (to assess body water), and dual-energy X-ray absorptiometry (DXA) [5]. DXA provides an estimation of the amount and regional distribution of FFM, LBM, FM, and bone mass [7]. DXA assessments are commonly used to determine the characteristics of a specific athlete group, to identify differences between athletes such as player position, or to track longitudinal changes of an individual athlete throughout a training cycle or calendar year [5].

There is a paucity of research reporting body composition characteristics of elite kayak athletes with initial data dating back to the 2000 Sydney Olympics, where 70 (50 men, 20 women) sprint kayak and canoeists underwent 38 anthropometry dimensions by trained anthropometrists [4]. The calculation of proportionality indices and Phantom z-scores revealed that kayakers and canoeists had greater relative shoulder and chest breadths, large upper body girths (arms and chest), and very low skinfolds, indicating a muscular upper body yet lean physique. Previous reports of Olympic canoeists at the 1976 Montreal Olympics [4], while similar in stature to athletes at the 2000 Sydney Olympics, were approximately 5 kg lighter with a higher sum of skinfolds. The progression of the sport with associated developments in training and athlete selection, equipment modifications (i.e., narrower boats), and shorter events in international competitions (i.e., 200 m event) are likely factors that have influenced a generational change in physique characteristics over a 25-year period. Recently, in a group of male kayakers, while not different in height and body mass, professional level kayakers (age 27.1 y) were reported to be leaner with lower absolute and relative fat mass and higher absolute and relative LBM than junior (age 16.2 y) and U23 (age 20.2 y) kayakers [8]. Not surprisingly, professional level kayakers in this study displayed greater strength and power measurements compared to junior and U23 kayakers. Contemporary physique data of women kayakers is currently lacking.

There is a paucity of data reporting body composition characteristics of contemporary kayak athletes (particularly women) at different stages of development as well as seasonal and/or annual changes that occur in body composition over a defined period of time, such as an Olympic quadrennial. Furthermore, due to the global COVID-19 pandemic and associated travel and health restrictions, athletes and national sporting organizations encountered several challenges unique to the 2020 Tokyo Summer Olympic quadrennial. This included, but was not limited to, quarantine requirements, training restrictions, modified training, closures of training centers, and postponement of Olympic-qualifying events [9]. The impact of these modifications to training and competition on the body composition of any athletic group is unknown, yet still important to understand due to the continuing omnipresence of COVID-19 and the potential impact of future pandemic events.

Thus, the primary aim of this study was to compare body composition characteristics of Australian elite senior and under 23 (U23) sprint kayak athletes. A secondary aim was to assess the longitudinal body composition changes of five senior men elite sprint kayak athletes over the course of the COVID-19-interrupted 2020 Tokyo Summer Olympic quadrennial.

## 2. Materials and Methods

### 2.1. Subjects

A convenience sample of all elite senior and U23 Australian National team kayakers (*n* = 32: M = 20, W = 12) training at the Australian Institute of Sport (AIS) High-Performance Sprint Canoe Facility (Gold Coast, Australia) were recruited for this study. Data were collected between 2017 and 2021 (COVID-19-interrupted quadrennial). The earliest DXA assessment for each athlete (*n* = 32) was used for a cross-sectional analysis between U23 and senior kayak athlete groups categorized by Paddle Australia at that initial assessment. Five senior men athletes (*n* = 2 Olympic gold medalists) who trained at the facility over the data collection period and had repeat DXA scans were used to monitor longitudinal changes in body composition. Athletes provided preliminary informed consent via their Athlete Management System database, before written informed consent was obtained prior to the commencement of the study. Approval was granted by the Bond University Human Research Ethics Committee (0000015933).

### 2.2. Body Composition Measurements

At each scanning occasion, athletes reported to the laboratory well rested after an overnight fast and were instructed to void their bladder on arrival. Body mass was measured using electronic scales (WM204, Wedderburn, Brisbane, QLD, Australia) while stature was measured using a wall-mounted stadiometer (Harpenden stadiometer, Holtain Limited, Crymych, Pembs, UK). From 2018 onwards, hydration status was assessed via urine-specific gravity (USG, PAL-10S, Atago, Saitama, Japan) from the first waking urine sample on the morning of the DXA assessment. Athletes were classified as euhydrated if their sample was <1.020 [10]. Athletes were instructed to remove all metal items on their body and were positioned supine on the DXA scanning bed (Lunar Prodigy DXA machine, GE Healthcare, Chicago, IL, USA) with the use of positional aids at the hands and feet. Athletes underwent standard scans for body composition and whole-body bone mineral content and the reference population for each athlete was matched for age, sex, and weight through the standard Combined Geelong/Lunar database. Scans were performed in accordance with a standardized protocol [11] by several qualified technicians throughout the period from 2017 to 2021. Furthermore, routine quality control using the phantom spine block and quality assurance protocols were undertaken prior to each scan to mitigate technical error [11]. All scans were re-segmented by one technician in a standardized manner at the end of the data collection period. Data on the athletes FM, FFM, LBM, and regional body composition from the scans were entered into an Excel spreadsheet by one researcher and checked for accuracy and abnormality. Lean mass ratio (LMR) was calculated by dividing LBM (kg) by body mass (kg). Upper body:lower body lean mass ratio was calculated by dividing upper body LBM (kg; arm and trunk) by lower body LBM (kg; legs).

### 2.3. Statistical Analysis

Statistical analysis was performed using GraphPad Prism (version 9.3.0 (463), GraphPad Software Inc., La Jolla, CA, USA). Descriptive statistics were reported as mean ± SD unless otherwise specified. Normality was assessed visually using histograms and tested using Q-Q plots and Shapiro–Wilks test for all variables. Differences between U23 and senior team athletes were assessed using independent t-tests. One-way ANOVA with Tukey post-hoc comparison was used to assess changes over time for the athletes involved in the longitudinal study. Effect size was calculated using Cohen’s *d*, with 0.2, 0.5, and 0.8 considered small, moderate, and large thresholds, respectively [12]. Statistical significance was accepted at *p* < 0.05.

## 3. Results

### 3.1. Whole-Body Composition—Senior vs. U23 Classification

Senior men sprint kayak athletes were significantly heavier (Table 1; *p* = 0.017; 10.3 ± 1.9 kg, *d* = 1.23), had a higher body mass index (*p* = 0.032; 1.9 ± 0.4 kg/m^2^, *d* = 1.09), FFM (*p* = 0.008; 9.4 ± 1.5 kg, *d* = 1.39), and LBM (*p* = 0.008; 9.0 ± 1.4 kg, *d* = 1.40) compared to U23 men athletes. There were no differences observed between senior and U23 men sprint kayak athletes in height (*p* = 0.081; 4.5 ± 1.0 cm, *d* = 0.87), body fat percentage (*p* = 0.437; −1.1 ± 0.6%, *d* = 0.37), and LMI (*p* = 0.596; 0.008 ± 0.01, *d* = 0.25).

The higher body mass of senior compared with U23 sprint women kayak athletes was not statistically different despite a large effect size (Table 1; *p* = 0.187; 3.8 ± 1.6 kg, *d* = 0.83). Senior sprint women kayakers were significantly taller (*p* = 0.041: 7.3 ± 1.8 cm, *d* = 1.37), had lower body fat percentage (*p* = 0.011; −4.3 ± 0.8%, *d* = 1.82) and higher FFM (*p* = 0.042; 5.5 ± 1.4 kg, *d* = 1.36), LBM (*p* = 0.048; 5.1 ± 1.3 kg, *d* = 1.32), and LMI (*p* = 0.039; 0.321 ± 0.008, *d* = 1.39) compared to U23 women athletes.

### 3.2. Regional Body Composition—Senior vs. U23 Classification

Senior men sprint kayak athletes had significantly higher arm LBM (Table 2; *p* = 0.005; 1.7 ± 0.3 kg, *d* = 1.49), trunk LBM (*p* = 0.005; 4.8 ± 0.7 kg, *d* = 1.51), leg LBM (*p* = 0.041; 2.4 ± 0.5 kg, *d* = 1.03), and upper body arm + trunk LBM (*p* = 0.004, 6.5 ± 0.9 kg, *d* = 1.56) compared to U23 men athletes. There were no differences observed between senior and U23 men sprint kayak athletes in arm, trunk, or leg FM, or in upper body:lower body lean mass ratio (Table 2).

Senior women sprint kayak athletes had significantly higher leg LBM (Table 2; *p* = 0.018; 1.9 ± 0.4 kg, *d* = 1.65). Despite no significant differences in upper body arm, trunk, and arm + trunk LBM, there were large effect sizes between groups (Table 2; *d* = 0.94, *d* = 1.16, *d* = 1.23, respectively). No difference was observed in upper body:lower body ratio between senior and U23 women sprint kayak athletes (Table 2).

### 3.3. Whole-Body Composition—Longitudinal Analysis of Senior Men Kayak Athletes

**Figure 1:**
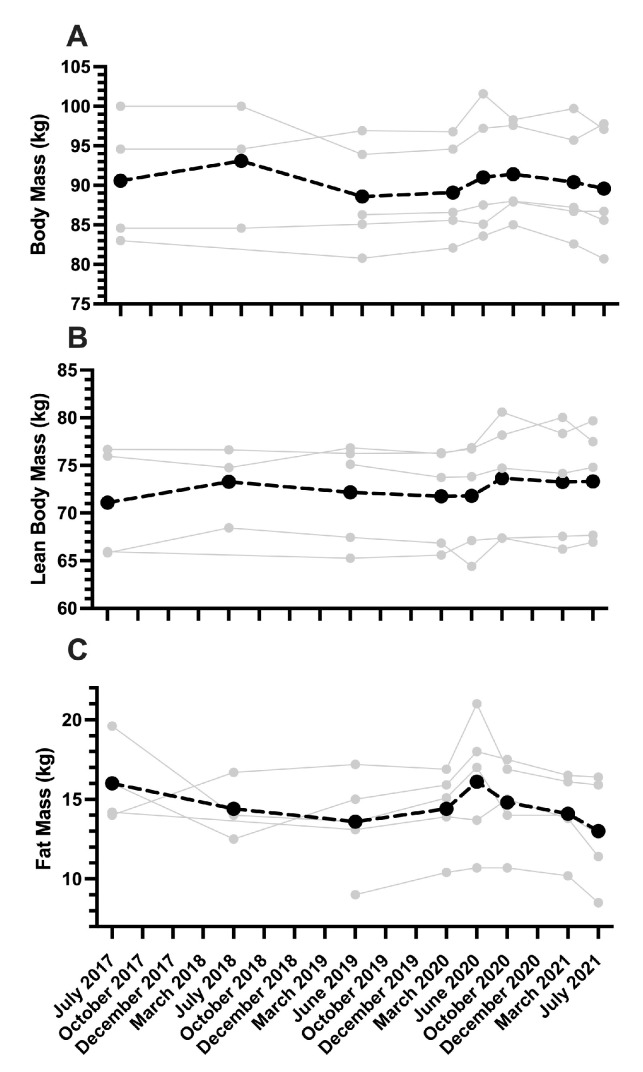
Body mass (**A**), lean body mass (**B**), and fat mass (**C**) of elite senior men sprint kayak athletes (*n* = 5) during the Tokyo 2020 Summer Olympic quadrennial. Body composition data were collected via DXA under standardized conditions. The light gray points represent individual athletes with the black dotted line indicating the group mean.

There was no significant difference between body mass at the beginning (July 2017) and end (July 2021) of the 2020 Tokyo Summer Olympic quadrennial (Figure 1A; July 2017: 90.6 ± 8.1 kg, July 2021: 90.4 ± 7.1 kg, *d* = 0.02) for senior men kayak athletes. Between March 2020 (following the postponement of the 2020 Tokyo Olympics) and October 2020, there was a significant increase in body mass (*p* = 0.022, MARCH 2020: 89.1 ± 6.3 kg, October 2020: 91.4 ± 6.1 kg, *d* = 0.04).

LBM was unchanged throughout the Olympic cycle (Figure 1B). A small, non-significant effect was observed for LBM between July 2017 and July 2021 (Figure 1B; *p* = 0.234; July 2017: 71.1 ± 6.0 kg, July 2021: 73.3 ± 5.8 kg, *d* = 0.38) and a similar small effect for LBM was observed between June 2020 and October 2020 (*p* = 0.284; June 2020: 71.8 ± 5.7 kg, October 2020: 73.6 ± 6.1 kg, *d* = 0.31).

Similarly, no change in FM was observed during the Olympic cycle (Figure 1C), but the effect size from July 2017 to July 2021 was large (Figure 1C; *p* = 0.774; July 2017: 16.0 ± 2.6 kg, July 2021: 13.0 ± 3.3 kg, *d* = 0.97). In the three-month period from March 2020 to June 2020, following the postponement of the 2020 Tokyo Olympics, the effect size was moderate but there were no significant differences observed in FM (*p* = 0.469, MARCH 2020: 14.4 ± 2.5 kg, June 2020: 16.1 ± 4.0 kg, *d* = 0.50). Then, from June 2020 to the pre-competition period in July 2021, no significant decrease in FM occurred, though the effect size was large between time-points (*p* = 0.070; June 2020: 16.1 ± 4.0 kg, July 2021: 13.0 ± 3.3 kg, *d* = 0.84).

## 4. Discussion

This study determined the body composition characteristics of Australian elite senior and U23 sprint kayakers and reported body composition changes of five senior men kayakers during the COVID-19-interrupted 2020 Tokyo Summer Olympic quadrennial. The primary findings were that senior men kayak athletes were heavier, had higher lean body mass (LBM), with similar fat mass (FM) percentages and regional distributions of FM in comparison to their U23 counterparts. In contrast, body mass was not significantly different between senior women compared with U23 kayak athletes, but senior women kayakers were taller, leaner (lower FM percentage), and had higher LBM and lean mass ratio (LMR) in comparison to U23 athletes.

There is paucity of published data on the body composition characteristics of contemporary elite men and women kayak athletes, with previous studies limited to junior national level women kayakers [13] and men and women senior kayakers from more than twenty years ago [4]. Recently, professional level men kayakers, while not different in height and body mass, were reported to be leaner and more muscular than junior and U23 kayakers [8]. In contrast, the elite men kayakers in the current study were significantly heavier, due to higher absolute LBM compared to their U23 counterparts. Similar differences have been reported in other sports including beach handball [14], rugby league [15], and Australian rules football [6]. Furthermore, senior men kayakers had similar FM and LMI compared to their U23 counterparts, indicating the development of higher LBM in senior athletes occurred while relative fat percentage remained stable. Regional analysis of the men kayakers revealed similar differences to those observed between senior and junior rugby league players, with senior athletes having higher LBM in the arms, trunk, and legs [15]. Unsurprisingly, higher LBM in professional level kayakers has been associated with greater strength and power outputs [8]. In a kayak-specific scenario, a 120 s maximal ergometer test was positively correlated with several physical characteristics including body mass, LBM in the arms, trunk, and legs [16]. Performance metrics were not collected in the current study, which limits any conclusions regarding the likely benefits of a higher LBM observed in men senior kayak athletes. Regardless, while the differences between athlete groups could be dismissed as the natural consequence of a more prolonged training history and physical maturation, our data can be a catalyst to establishing the rate and magnitude of body composition change from youth to senior transition so that strategies may be improved to promote elite athlete development.

Senior women kayakers had a higher LBM compared to U23 women kayakers, yet body mass was not different. Accordingly, the observed differences in body composition in U23 women kayakers were explained by a higher FM percentage and lower LMI in comparison to their senior counterparts. FM accumulates more readily in young women compared to young men due to increases in estrogen during puberty and early adulthood [17] which may partially explain the higher percentage in FM observed in the U23 women kayakers in this study. Increased training load and associated energy expenditure, as well as greater exposure to resistance training in senior kayak athletes, could be a primary explanation why body fat percentage was lower and lean mass was higher in senior kayakers. In young (13.6 years) Spanish women kayakers (*n* = 86), the most successful (Top 10) athletes were older and more mature, and had a higher percentage of muscle mass than other competitors [12], highlighting the importance of physique development. However, it is difficult to undertake the longitudinal measurement of sex hormones, body composition, and the training and performance variables needed to determine the multifactorial associations, or cause and effect, that may explain the successful transition from junior to elite women sprint kayakers. Clearly, further work is needed to understand the longitudinal changes in body composition for women sprint kayakers and if this differs from the longitudinal changes observed in men. For now, our data indicate that LBM appears to differentiate between women’s kayak levels and could be a focal point for future athlete development.

Across the Tokyo 2017–2021 Olympic quadrennial, there were no significant differences in any body composition variable for the five elite men kayakers. The limited number of senior kayak athletes competing at an international level throughout the quadrennial limited the sample of the current study. Therefore, the one-way ANOVA analysis was likely underpowered, which is a common limitation in research conducted on elite athlete cohorts, and emphasis on the magnitude of change rather than statistical significance is recommended for this population [18]. Assessing effect sizes shows that FM decreased; there was a small increase in LBM, whilst body mass remained stable for elite men kayakers across the quadrennial. To our knowledge, this is the first time longitudinal changes in body composition have been reported for elite kayak athletes across an entire Olympic cycle. There was a moderate increase in FM between March 2020 and July 2020 after the postponement of the Tokyo Olympics until 2021. This increase in FM presumably occurred due to the decrease in exercise energy expenditure caused by the eight-week closure of the high-performance kayak training facility due to national COVID-19 restrictions and cessation of planned training during this time. After training resumed, FM decreased (July 2020 to October 2020) to a similar level as March 2020, showcasing the dynamic response of fat tissue to increases and decreases in exercise energy expenditure and/or change in dietary and lifestyle habits. In contrast, only small changes in LBM occurred across the Olympic quadrennial. It is known that the muscle hypertrophy response for strength-trained men is less than non-strength-trained men [19]. Therefore, the high upper body strength and power and prolonged training history of elite men senior kayakers may reduce the capacity for ongoing hypertrophy across the quadrennial. The largest increase in LBM throughout the cycle was observed between June and October 2020 when the kayakers returned to normal training after the COVID-19 hiatus. Since athletes had no upcoming international competition, an extensive strength training block with an expected concomitant increase in LBM occurred during this time due to a longer-than-anticipated Olympic macro-cycle. Despite increased strength training, only a small increase in LBM occurred, suggesting that LBM gains are difficult to stimulate for elite senior kayakers. Amongst the five individual athletes who were tracked longitudinally, two were Olympic gold medal winners at the Tokyo 2020 Olympic Games. Importantly, these two athletes had a higher LBM compared to the average of the group. This supports the notion that a higher body mass and LBM, as seen in this modest group of men senior elite kayakers and Olympic medalists, is associated with improved senior kayaking performance outcomes.

Despite our best efforts, no women athletes met the inclusion criteria for the longitudinal analysis due to limited data caused by changes in squad selection and restricted availability of athletes for data collection due to training and competition schedules. Future longitudinal studies in elite kayakers must include women kayakers to provide new insight for longitudinal body composition changes and any sex-based differences. We acknowledge that our failure to accurately capture and subsequently report training load and dietary intake across the data capture period is a limitation of the current study. Longitudinal training data and dietary intake data would provide further insight into understanding the body composition changes that occurred in the five men athletes across the Olympic quadrennium. Future studies reporting longitudinal changes in body composition should include training data and dietary periodization strategies to determine the effects of changes in training and diet on body composition. Future research, which describes the training load and dietary intake of U23 and senior kayakers and the relationship between these variables and body composition, would allow practitioners to plan training and nutritional interventions, which would in turn benefit their performance at an U23 and senior kayak competition level, respectively.

### Practical Application

The findings from this study indicate that an important characteristic for men sprint kayak athletes transitioning from U23 to senior ranks is to increase body mass. Athletes should engage in resistance training sessions and be well resourced with appropriate support staff to implement dietary strategies that promote muscle protein synthesis and create an energy surplus to increase body mass [20]. Incorporating resistance training throughout the annual training plan while maintaining an appropriate energy surplus is pivotal to achieving a favorable change in body mass for men kayak athletes as they transition to senior training and competition levels. In contrast, while similar in body mass, senior kayak women athletes are leaner (lower percent body fat) than their younger counterparts. Acknowledging that resistance training is incorporated throughout the annual training plan for female kayak athletes, practitioners should carefully consider dietary strategies to optimize increases in lean body mass while maintaining energy balance [21]. Understanding the differences that exist in body composition characteristics of U23 and senior men and women kayak athletes will assist practitioners in designing future training programs and dietary interventions.

## 5. Conclusions

In conclusion, senior men kayak athletes have greater total body mass, yet are proportionally similar to younger kayak athletes in terms of body composition (i.e., similar body fat percentage). In contrast, senior and U23 women are similar in body mass but differ in body composition, as senior women kayak athletes have a higher LBM and lower body fat percentage compared to younger U23 athletes. These differences are important considerations when planning training and diet interventions for men and women kayak athletes throughout their development. Regarding the longitudinal monitoring of the five elite senior men kayak athletes, there was no significant changes in body composition across an Olympic quadrennial. However, the cessation of training during the COVID-19 pandemic resulted in moderate fluctuations in FM and LBM. This change is not surprising and likely represents a training load and dietary intake modification, but the metabolic flexibility of highly trained athletes appears to limit the impact on body composition long term.

## Figures and Tables

**Table 1:** Whole-body composition characteristics of elite men and women senior and U23 sprint kayak athletes. Data were collated from the earliest initial body composition assessment during 2017 (July) for each athlete and are presented as mean ± SD.

	Men (*n* = 20)		Women (*n* = 12)	
	Senior (*n* = 13)	U23 (*n* = 7)	Senior (*n* = 5)	U23 (*n* = 7)
Age (years)	24.7 ± 5.0 ***	18.6 ± 1.2	26.0 ± 3.2 ^a^**	20.0 ± 1.8
Height (m)	1.86 ± 0.05 ^a^	1.81 ± 0.05	1.76 ± 0.06 *^a^	1.69 ± 0.05
Mass (kg)	88.6 ± 7.1 *^a^	78.2 ± 10.6	71.1 ± 5.7 ^a^	67.3 ± 3.7
BMI (kg·m^−2^)	25.6 ± 1.4 *^a^	23.7 ± 2.3	22.9 ± 1.0	23.7 ± 1.5
Fat Mass (kg)	14.1 ± 3.2	13.4 ± 3.0	14.1 ± 1.7 *^a^	16.3 ± 1.7
Fat Mass (%)	16.5 ± 3.3	17.6 ± 1.7	20.8 ± 2.8 *^a^	25.0 ± 2.0
Lean Body Mass (kg)	71.3 ± 6.1 **^a^	62.3 ± 7.1	53.8 ± 5.3 *^a^	48.7 ± 2.5
Lean Mass Ratio	0.81 ± 0.03	0.80 ± 0.02	0.76 ± 0.03 *^a^	0.72 ± 0.02
FFM (kg)	74.9 ± 6.3 **^a^	65.5 ± 7.4	56.7 ± 5.5 *^a^	51.3 ± 2.6
BMC (g)	3541 ± 299 *	3234 ± 361	2878 ± 306 *^a^	2545 ± 167

BMI, body mass index; FFM, fat free mass; BMC, bone mineral content. * *p* < 0.05, ** *p* < 0.01, *** *p* < 0.001, ^a^ = large effect size (Cohen’s *d* > 0.8).

**Table 2:** Regional body composition characteristics of elite men and women senior and U23 sprint kayak athletes. Data were collated from the earliest initial body composition assessment during 2017 (July) for each athlete and are presented as mean ± SD.

		Men (*n* = 20)		Women *n* = 12	
		Senior (*n* = 13)	(U23 *n* = 7)	(Senior *n* = 5)	(U23 *n* = 7)
Arms	Lean Mass (kg)	10.1 ± 1.1 **^a^	8.4 ± 1.2	6.5 ± 0.5 ^a^	6.0 ± 0.6
	Fat Mass (kg)	1.6 ± 0.3	1.5 ± 0.2	1.5 ± 0.1 *	1.9 ± 0.2
Trunk	Lean Mass (kg)	36.4 ± 3.1 **^a^	31.7 ± 3.3	28.4 ± 3.5 ^a^	25.5 ± 1.2
	Fat Mass (kg)	6.2 ± 1.8	5.7 ± 2.1	5.5 ± 1.1 ^a^	6.8 ± 1.1
Legs	Lean Mass (kg)	21.3 ± 2.1 *^a^	19.0 ± 2.7	16.0 ± 1.4 *^a^	14.1 ± 0.9
	Fat Mass (kg)	5.4 ± 1.2	5.4 ± 0.9	6.2 ± 0.8	6.8 ± 1.1
Upper Body	Mass (kg)	56.0 ± 4.5 **^a^	48.7 ± 6.7	43.2 ± 3.7	41.3 ± 2.3
	Lean Mass (kg)	46.5 ± 4.0 **^a^	40.1 ± 4.4	34.9 ± 3.9 ^a^	31.5 ± 1.5
	Upper Body:Lower Body Lean Mass Ratio	2.18 ± 0.11	2.12 ± 0.10	2.17 ± 0.12	2.24 ± 0.10

* *p* < 0.05, ** *p* < 0.01, ^a^ = large effect size (Cohen’s d >0.8).

## Data Availability

The raw data supporting the conclusions of this article will be made available by the authors on request. Due to the small sample size of elite kayakers, to ensure participant confidentiality, individual participant age will not be provided.
